# Supplementary figures and images for: Improving access to care and community health in Haiti with optimized community health worker placement
Source: PLOS Glob Public Health. 2022 May 10;2(5):e0000167. doi: 10.1371/journal.pgph.0000167 (PMC10022239; doi:10.1371/journal.pgph.0000167)

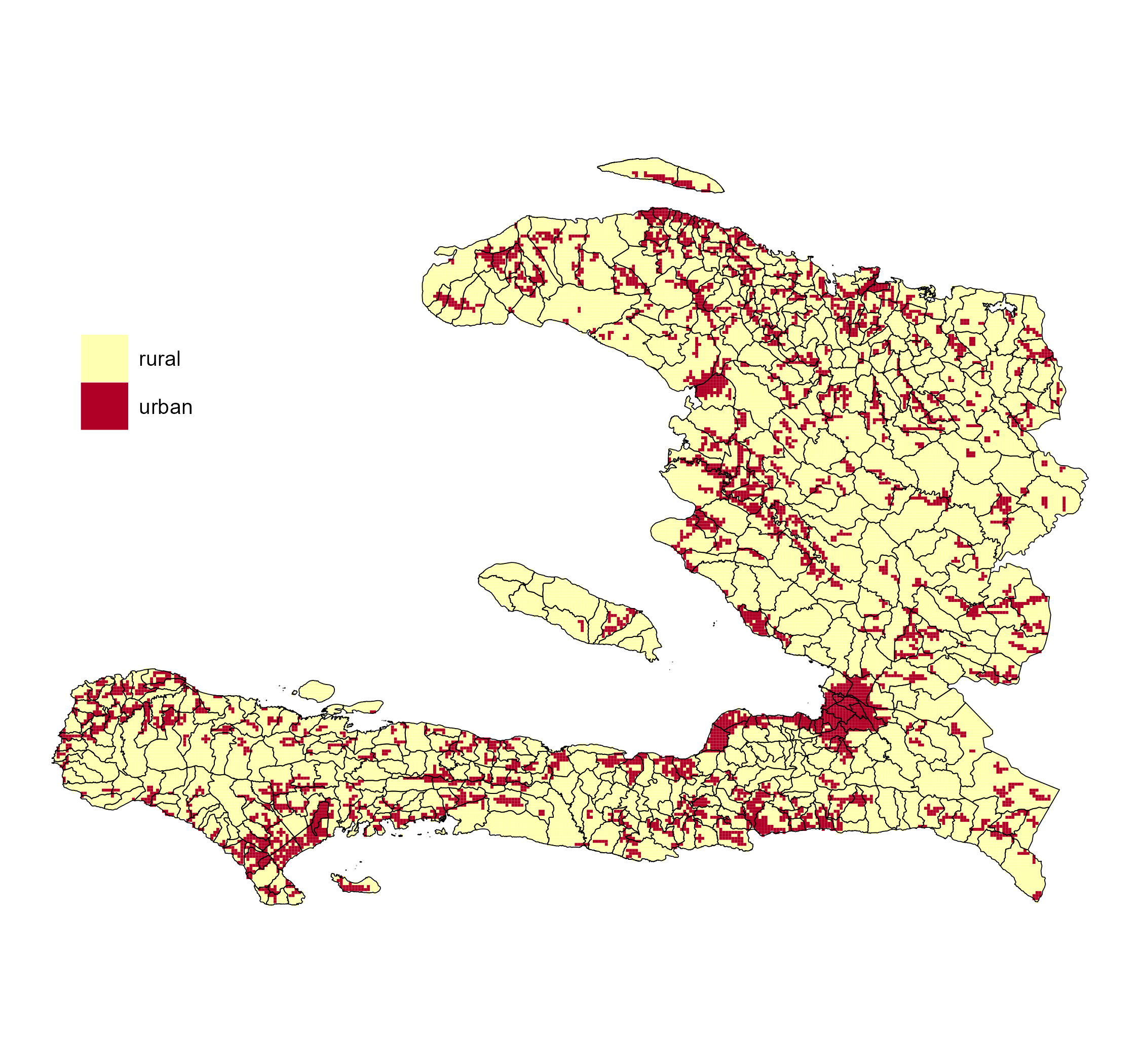

Supplement: S1 Fig — Calculated following the European Commission’s “degree of urbanization approach” described in [43] based on predictions of population density in 2020 per square kilometre [27,29]. The shapefile from the Centre National de l’Information Géo-Spatiale (CNIGS) was used [30] (available at https://data.humdata.org/dataset/hti-polbndl-adm1-cnigs-zip). (TIFF) [file pgph.0000167.s001.tiff]

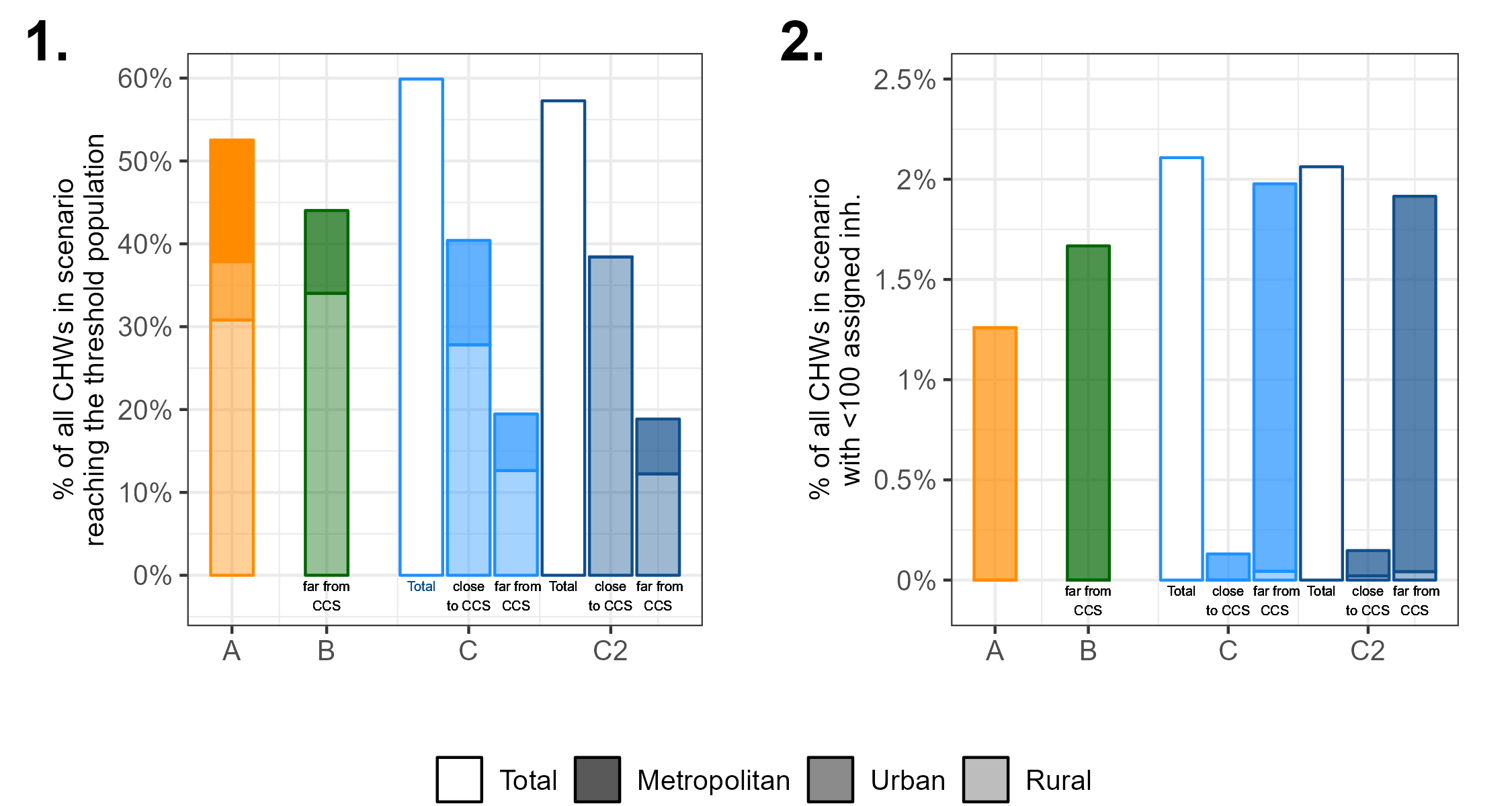

Supplement: S2 Fig — 1. Percentage of CHWs in each scenario who are assigned to the threshold number of inhabitants as defined in Fig 1 and S2 Table. 2. Percentage of CHWs in each scenario who are assigned to less than 100 inhabitants. The metropolitan area is only defined for scenario A; in scenario B, the travel time defining areas close to a community health centre (CCS) is 30 minutes; in scenarios C and C2, it is 60 minutes (cf. Fig 1). (TIFF) [file pgph.0000167.s002.tiff]

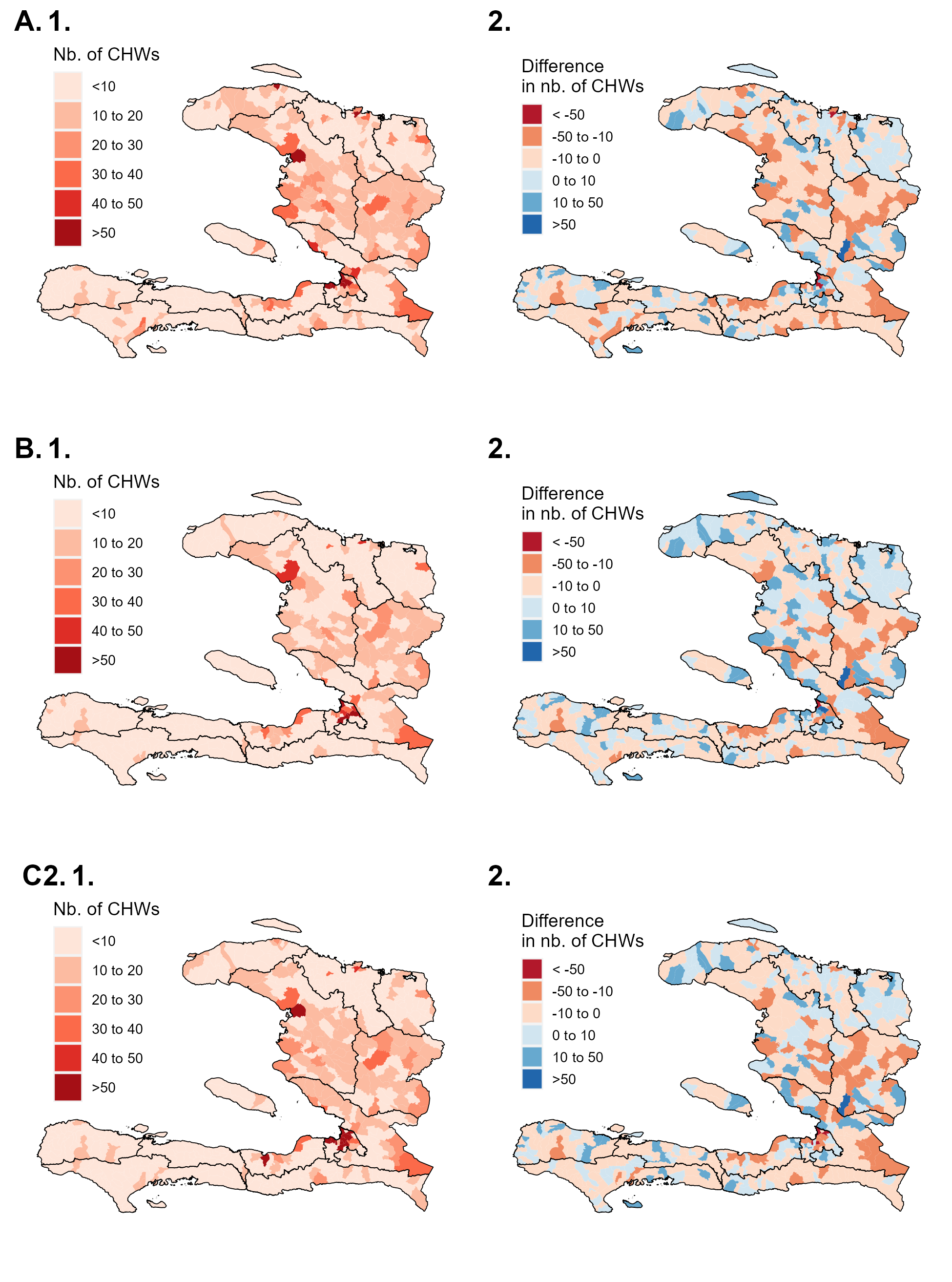

Supplement: S3 Fig — 1. Number of CHWs per section communale, according to scenarios A, B or C2 respectively. 2. Difference by section communale between the current number of CHWs in the SPA survey and the suggested number of CHWs under the scenarios A, B or C2 respectively. Negative values (signified in red) indicate a deficit, positive values (in blue) signify a surplus. The shapefile from the Centre National de l’Information Géo-Spatiale (CNIGS) was used [30] (available at https://data.humdata.org/dataset/hti-polbndl-adm1-cnigs-zip). (TIFF) [file pgph.0000167.s003.tiff]

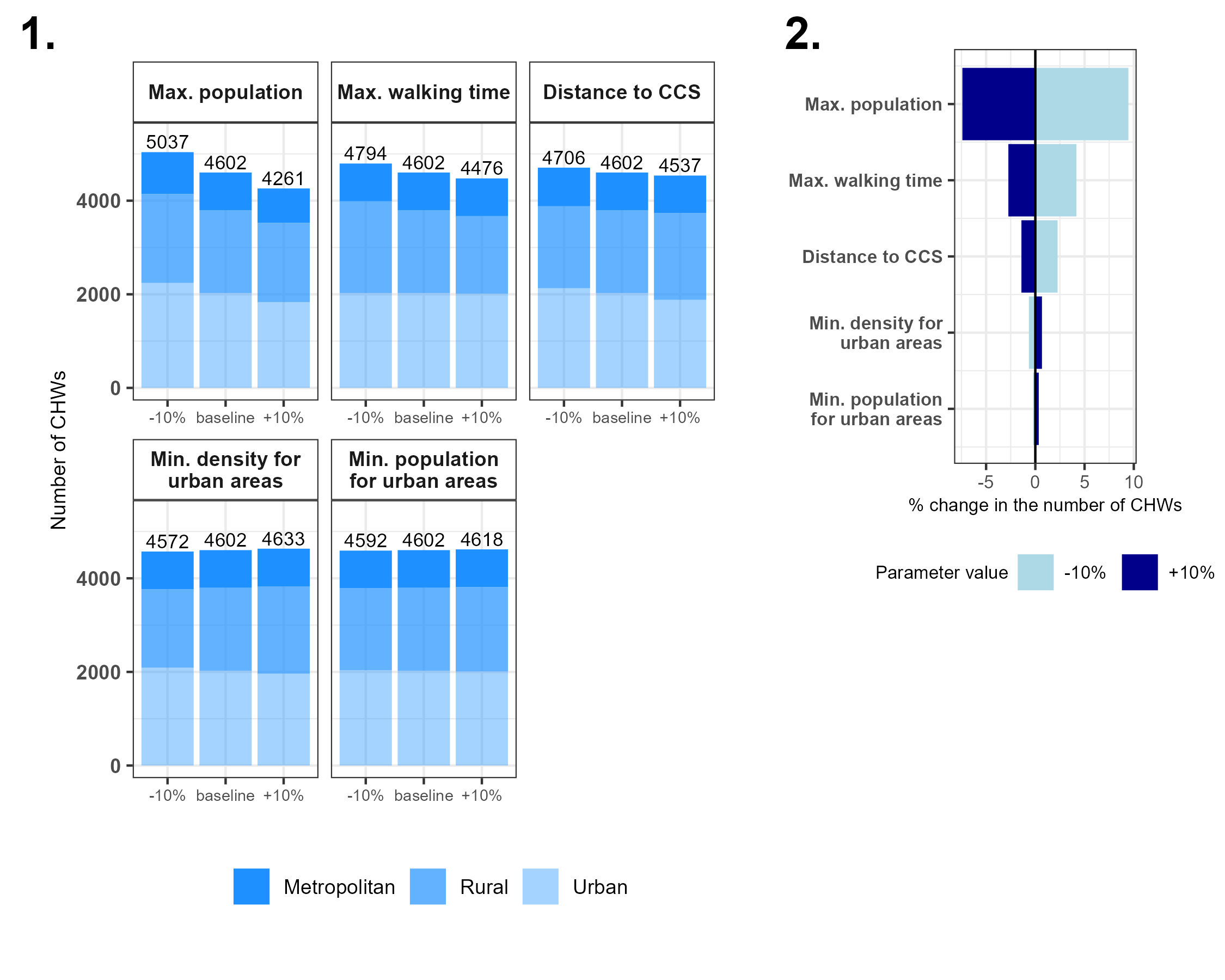

Supplement: S4 Fig — 1. Required number of CHW at the national level. 2. Percent increase or decrease in national number of CHWs required if parameter values are increased or decreased by 10%. (TIFF) [file pgph.0000167.s004.tiff]

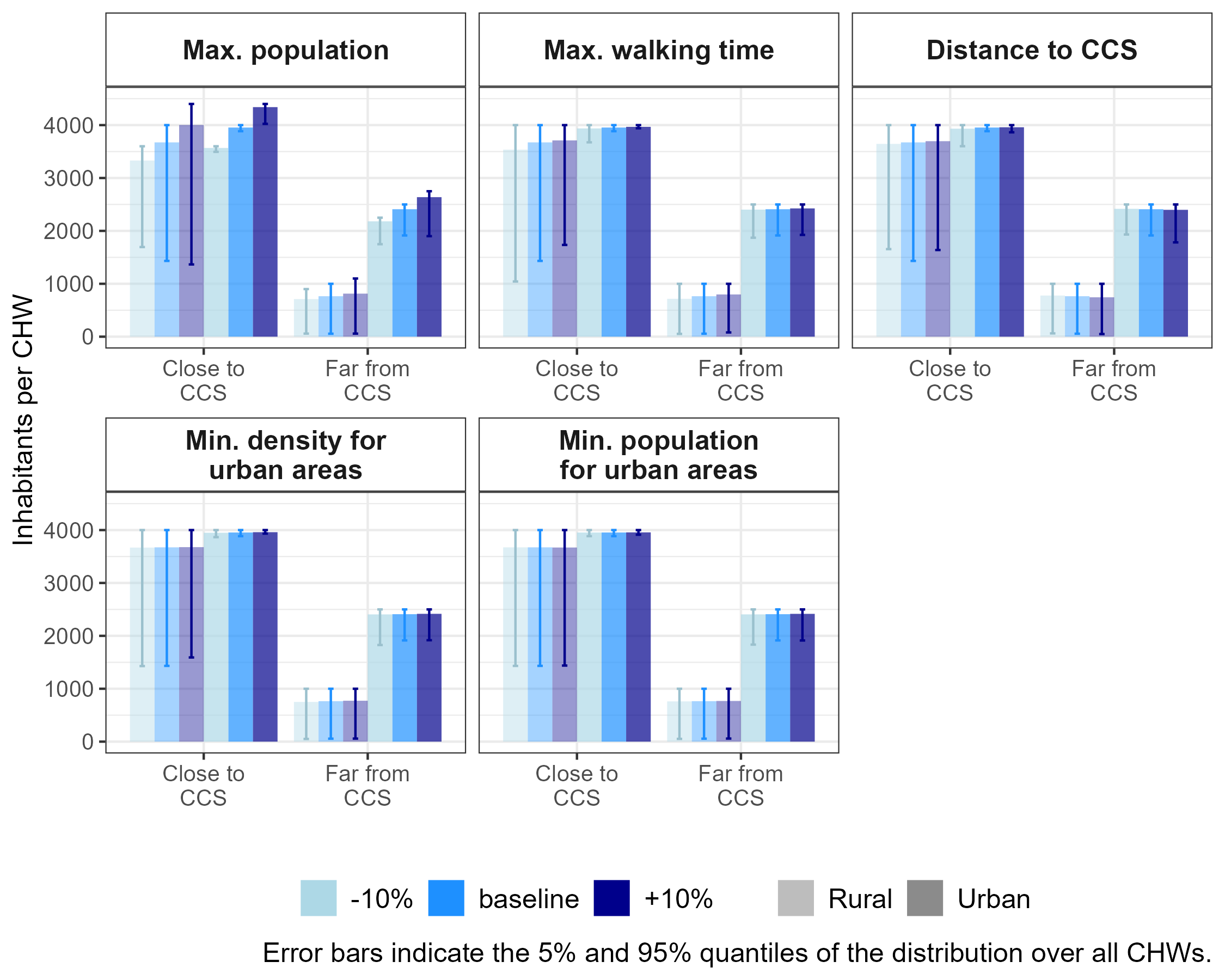

Supplement: S5 Fig — (TIFF) [file pgph.0000167.s005.tiff]

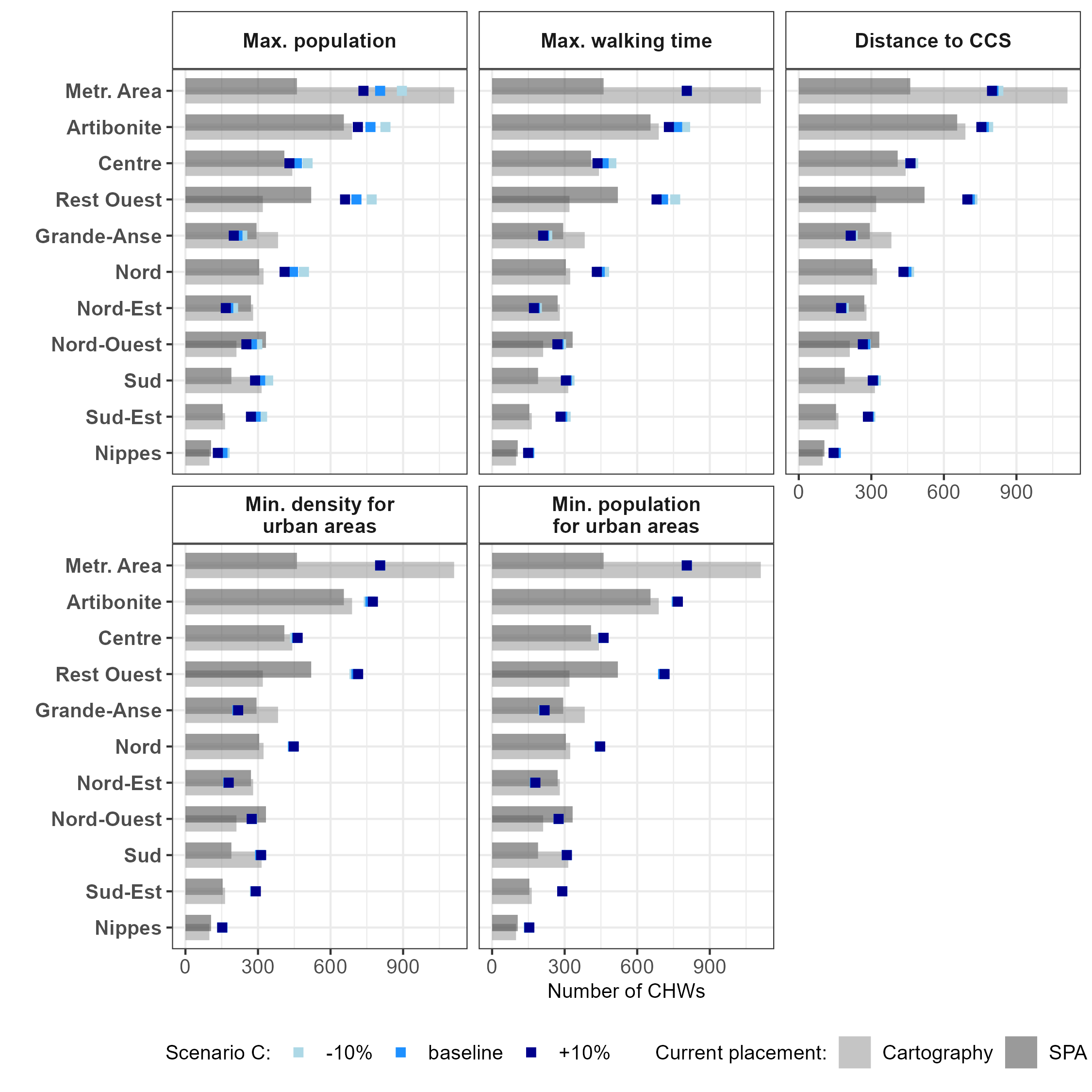

Supplement: S6 Fig — (TIFF) [file pgph.0000167.s006.tiff]
