# Supplementary material for: Improving access to care and community health in Haiti with optimized community health worker placement
Source: PLOS Glob Public Health. 2022 May 10;2(5):e0000167. doi: 10.1371/journal.pgph.0000167 (PMC10022239; doi:10.1371/journal.pgph.0000167)
Supplement: S1 Table — (PDF) [file pgph.0000167.s007.pdf]

Number of visits	Threshold calculation	Value used
2 visits per year	8*4.3*220/2=3784	4000
3 visits per year	8*4.3*220/3=2523	2500
8 visits per year	8*4.3*220/8=946	1000

S1 Table. Assumed correspondence between the number of visits per year and the threshold on the number of inhabitants assigned per CHW.
