# Supplementary material for: Improving access to care and community health in Haiti with optimized community health worker placement
Source: PLOS Glob Public Health. 2022 May 10;2(5):e0000167. doi: 10.1371/journal.pgph.0000167 (PMC10022239; doi:10.1371/journal.pgph.0000167)
Supplement: S2 Table — CCS refers to community health centres (centres communautaires de santé). L denotes the set of locations containing a CCS, J the potential CHW locations. Additional notations are provided in the main text. (PDF) [file pgph.0000167.s008.pdf]

Scenario	Maximum walking time *(S)*	**Maximum number of people *(k_j)***		***Locations (J, I)***
**A**	60 min.	Non-metropolitan area	- 1000 in rural areas<br>- 2500 in urban areas	$J = I = \{i \mid \mathrm{a_i} > 0\}$
		Metropolitan area	- 4000	
**B**	60 min.	$\geq$ 30 min walk from a CCS	- 1000 in rural areas<br>- 2500 in urban areas	$J = I = \{i \mid \mathrm{a_i} > 0, \min_{l \in L} d_{il} \geq 30\ \mathrm{min}\}$
		<30 min walk from a CCS	None	$J = I = \emptyset$
**C**	60 min.	$\geq$ 60 min walk from a CCS	- 1000 in rural areas,<br>- 2500 in urban areas	$J = I = \{i \mid \mathrm{a_i} > 0, \min_{l \in L} d_{il} \geq 60\ \mathrm{min}\}$
		<60 min walk from a CCS	- 4000	$J = I = \{i \mid \mathrm{a_i} > 0, \min_{l \in L} d_{il} < 60\ \mathrm{min}\}$
**C2**	60 min.	$\geq$ 60 min walk from a CCS	- 1000 in rural areas<br>- 2500 in urban areas	$J = I = \{i \mid \mathrm{a_i} > 0, \min_{l \in L} d_{il} \geq 60\ \mathrm{min}\}$
		<60 min walk from a CCS	- 1000 in rural areas<br>- 4000 in urban areas	$J = I = \{i \mid \mathrm{a_i} > 0, \min_{l \in L} d_{il} < 60\ \mathrm{min}\}$

S2 Table. Description of the four scenarios selected. CCS refers to Community Health Centres (*Centres communautaires de santé*). L denotes the set of locations containing a CCS , J the potential CHW locations. Additional notations are provided in the main text.
