# Supplementary material for: Improving access to care and community health in Haiti with optimized community health worker placement
Source: PLOS Glob Public Health. 2022 May 10;2(5):e0000167. doi: 10.1371/journal.pgph.0000167 (PMC10022239; doi:10.1371/journal.pgph.0000167)
Supplement: S1 Text — (PDF) [file pgph.0000167.s009.pdf]

S1 Text. Presentation of the four scenarios per department

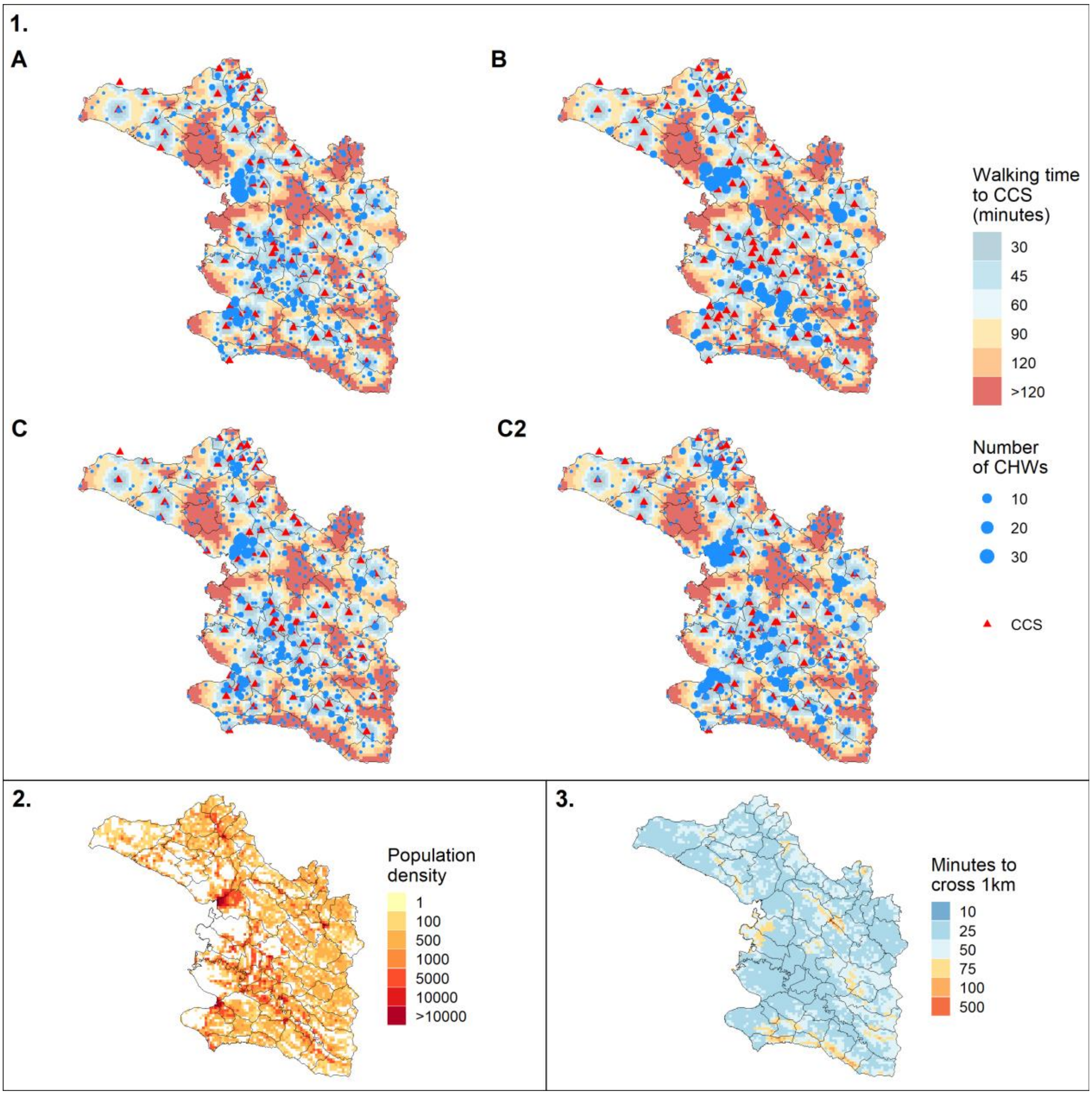

S1 Text Fig 1. Comparison of the placement scenarios in the Artibonite department. The four CHW placement scenarios (A, B, C and C2). CHW positions are indicated with blue dots, community health centres (CCS) are indicated with red triangles. The colored surface indicates the predicted walking time to the closest CCS using the methodology by Weiss and colleagues (1,2): difficult-to-reach areas, located more than 60 minutes walk from the nearest CCS, are shown in orange/red and areas with easier access (less than 60 minutes walk) are shown in blue. 2. For interpretability: prediction of population density in 2020 per square kilometre (3,4). 3. For interpretability: walking time friction surface by Weiss et al. is shown as the time required to cross 1km (1). The shapefile from the Centre National de l'Information Géo-Spatiale (CNIGS) was used (5) (available at https://data.humdata.org/dataset/hti-polbndl-adm1-cnigs-zip).

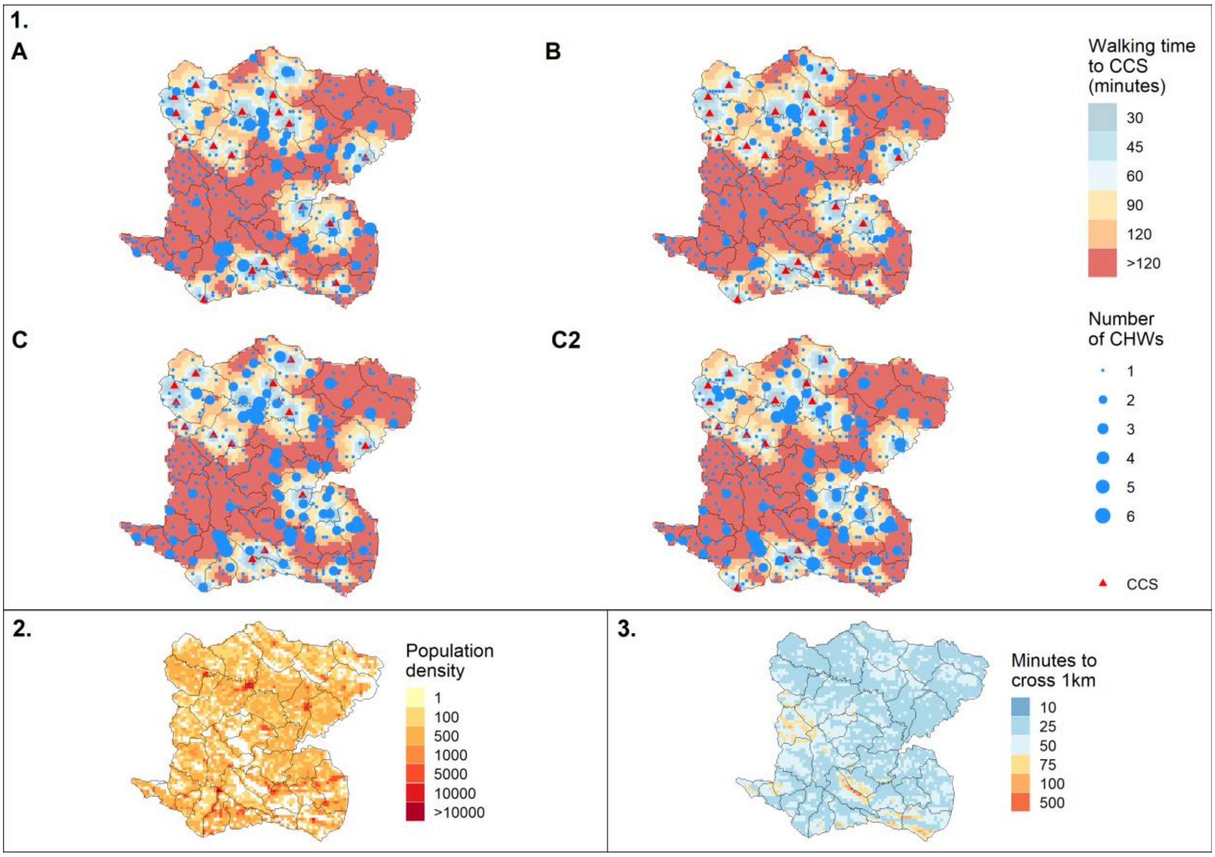

S1 Text Fig 2. Comparison of the placement scenarios in the Centre department. The four CHW placement scenarios (A, B, C and C2). CHW positions are indicated with blue dots, community health centres (CCS) are indicated with red triangles. The colored surface indicates the predicted walking time to the closest CCS using the methodology by Weiss and colleagues (1,2): difficult-to-reach areas, located more than 60 minutes walk from the nearest CCS, are shown in orange/red and areas with easier access (less than 60 minutes walk) are shown in blue. 2. For interpretability: prediction of population density in 2020 per square kilometre (3,4). 3. For interpretability: walking time friction surface by Weiss et al. is shown as the time required to cross 1km (1). The shapefile from the Centre National de l'Information Géo-Spatiale (CNIGS) was used (5) (available at https://data.humdata.org/dataset/hti-polbndl-adm1-cnigs-zip).

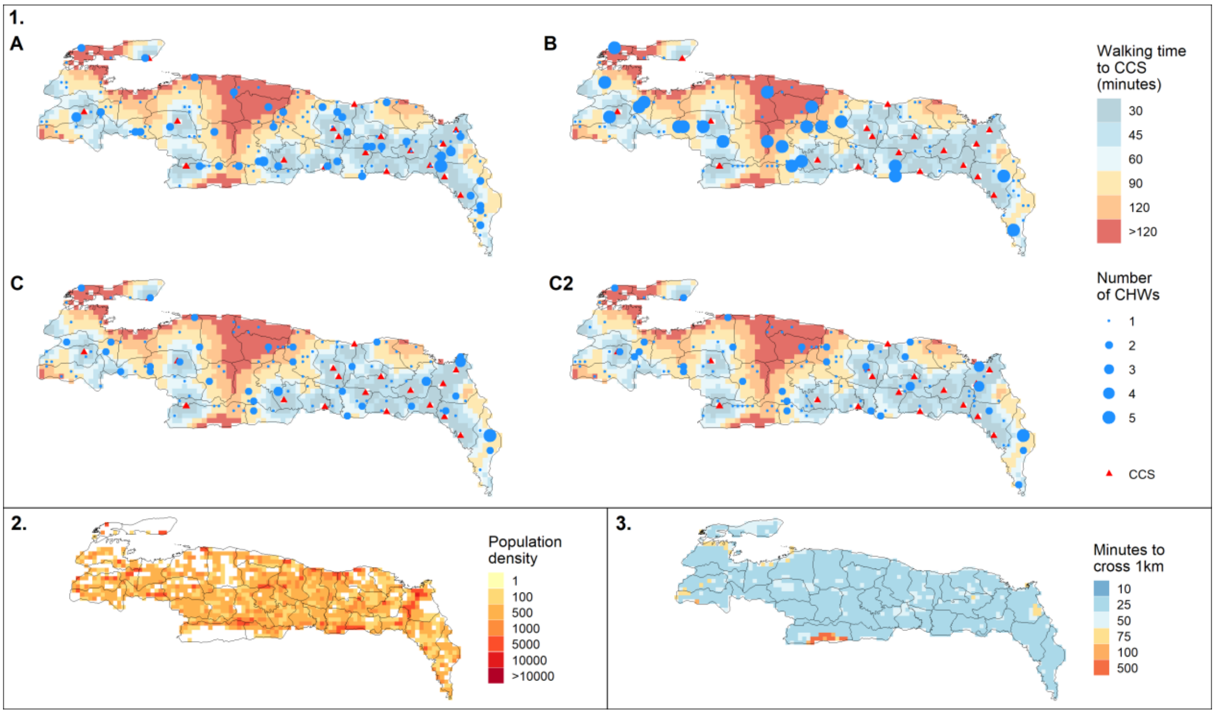

S1 Text Fig 3. Comparison of the placement scenarios in the Nippes department. The four CHW placement scenarios (A, B, C and C2). CHW positions are indicated with blue dots, community health centres (CCS) are indicated with red triangles. The colored surface indicates the predicted walking time to the closest CCS using the methodology by Weiss and colleagues (1,2): difficult-to-reach areas, located more than 60 minutes walk from the nearest CCS, are shown in orange/red and areas with easier access (less than 60 minutes walk) are shown in blue. 2. For interpretability: prediction of population density in 2020 per square kilometre (3,4). 3. For interpretability: walking time friction surface by Weiss et al. is shown as the time required to cross 1km (1). The shapefile from the Centre National de l'Information Géo-Spatiale (CNIGS) was used (5) (available at https://data.humdata.org/dataset/hti-polbndl-adm1-cnigs-zip).

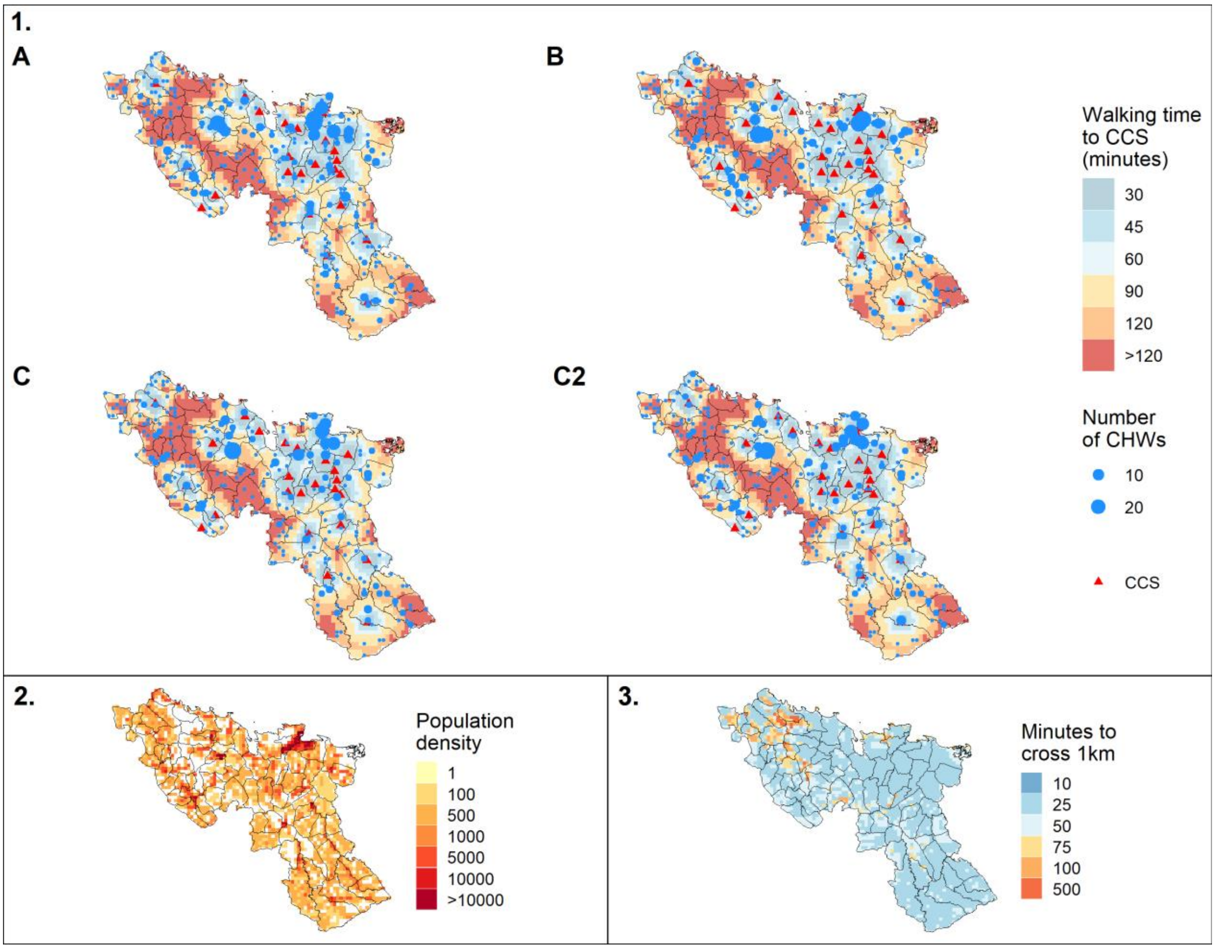

S1 Text Fig 4. Comparison of the placement scenarios in the Nord department. The four CHW placement scenarios (A, B, C and C2). CHW positions are indicated with blue dots, community health centres (CCS) are indicated with red triangles. The colored surface indicates the predicted walking time to the closest CCS using the methodology by Weiss and colleagues (1,2): difficult-to-reach areas, located more than 60 minutes walk from the nearest CCS, are shown in orange/red and areas with easier access (less than 60 minutes walk) are shown in blue. 2. For interpretability: prediction of population density in 2020 per square kilometre (3,4). 3. For interpretability: walking time friction surface by Weiss et al. is shown as the time required to cross 1km (1). The shapefile from the Centre National de l'Information Géo-Spatiale (CNIGS) was used (5) (available at https://data.humdata.org/dataset/hti-polbndl-adm1-cnigs-zip).

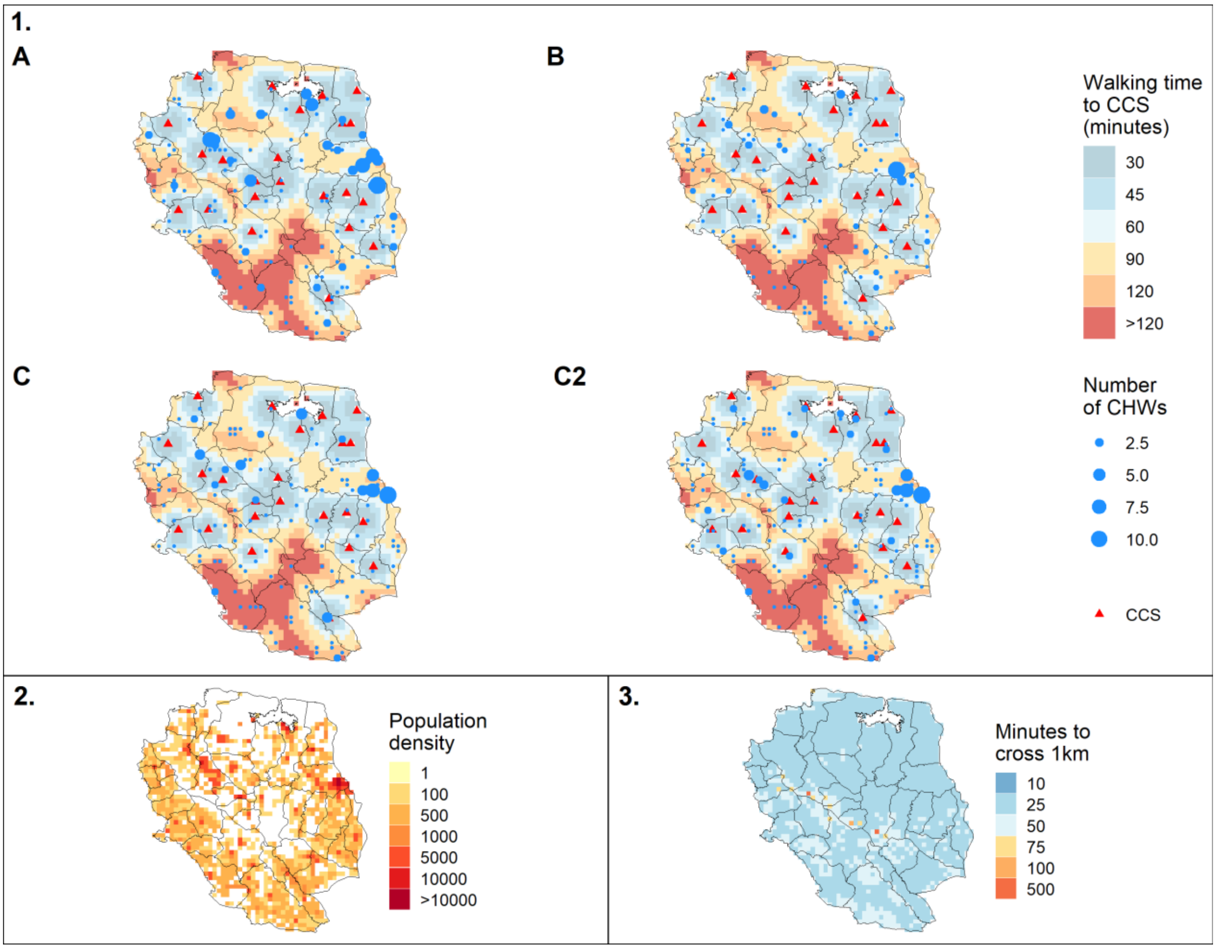

S1 Text Fig 5. Comparison of the placement scenarios in the Nord-Est department. The four CHW placement scenarios (A, B, C and C2). CHW positions are indicated with blue dots, community health centres (CCS) are indicated with red triangles. The colored surface indicates the predicted walking time to the closest CCS using the methodology by Weiss and colleagues (1,2): difficult-to-reach areas, located more than 60 minutes walk from the nearest CCS, are shown in orange/red and areas with easier access (less than 60 minutes walk) are shown in blue. 2. For interpretability: prediction of population density in 2020 per square kilometre (3,4). 3. For interpretability: walking time friction surface by Weiss et al. is shown as the time required to cross 1km (1). The shapefile from the Centre National de l'Information Géo-Spatiale (CNIGS) was used (5) (available at https://data.humdata.org/dataset/hti-polbndl-adm1-cnigs-zip).

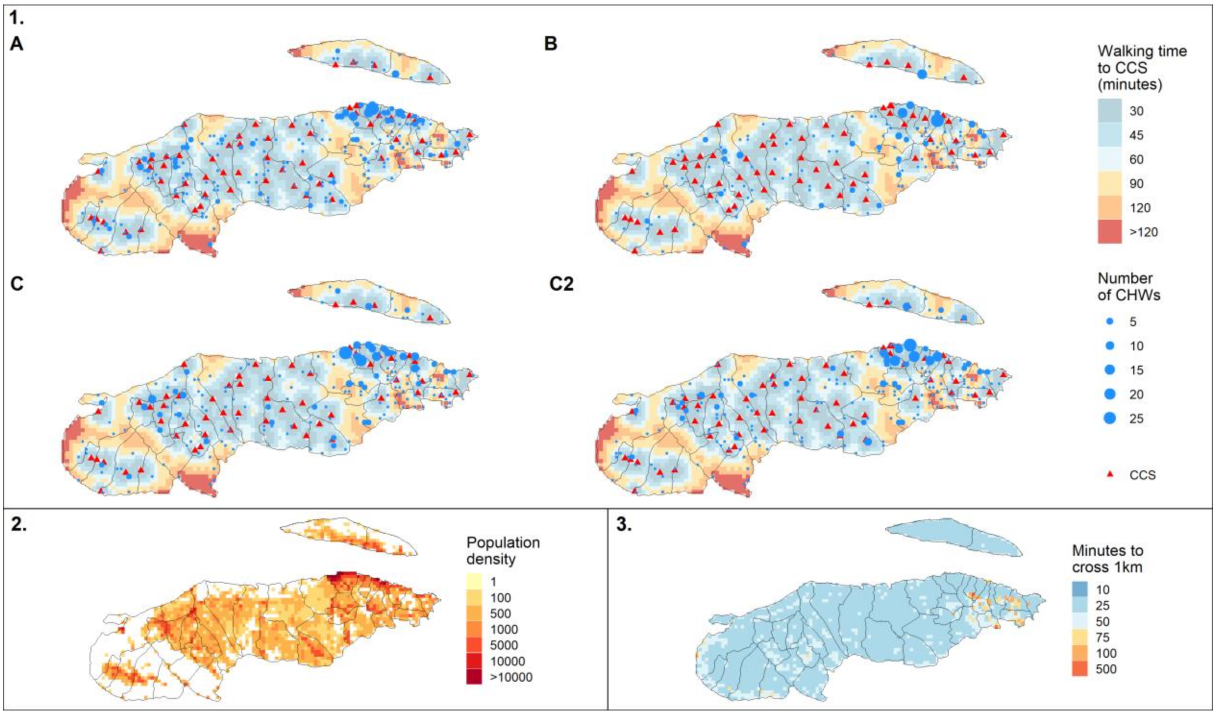

S1 Text Fig 6. Comparison of the placement scenarios in the Nord-Ouest department. The four CHW placement scenarios (A, B, C and C2). CHW positions are indicated with blue dots, community health centres (CCS) are indicated with red triangles. The colored surface indicates the predicted walking time to the closest CCS using the methodology by Weiss and colleagues (1,2): difficult-to-reach areas, located more than 60 minutes walk from the nearest CCS, are shown in orange/red and areas with easier access (less than 60 minutes walk) are shown in blue. 2. For interpretability: prediction of population density in 2020 per square kilometre (3,4). 3. For interpretability: walking time friction surface by Weiss et al. is shown as the time required to cross 1km (1). The shapefile from the Centre National de l'Information Géo-Spatiale (CNIGS) was used (5) (available at https://data.humdata.org/dataset/hti-polbndl-adm1-cnigs-zip).

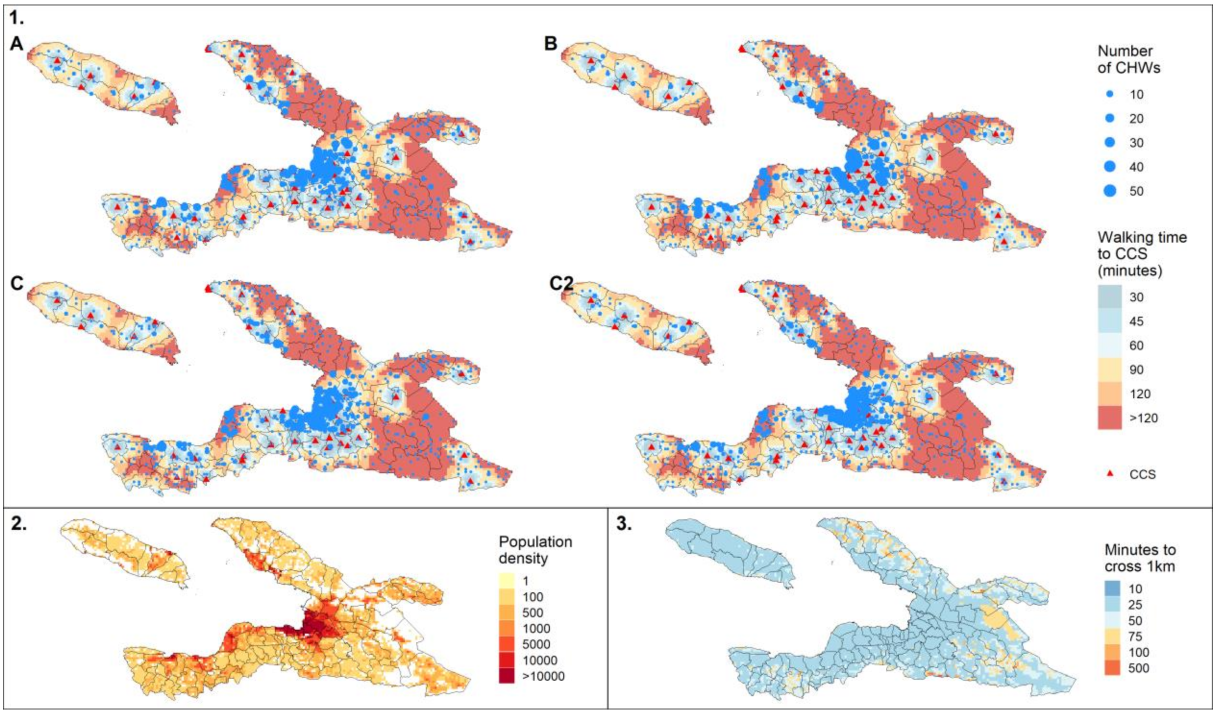

S1 Text Fig 7. Comparison of the placement scenarios in the Ouest department. The four CHW placement scenarios (A, B, C and C2). CHW positions are indicated with blue dots, community health centres (CCS) are indicated with red triangles. The colored surface indicates the predicted walking time to the closest CCS using the methodology by Weiss and colleagues (1,2): difficult-to-reach areas, located more than 60 minutes walk from the nearest CCS, are shown in orange/red and areas with easier access (less than 60 minutes walk) are shown in blue. 2. For interpretability: prediction of population density in 2020 per square kilometre (3,4). 3. For interpretability: walking time friction surface by Weiss et al. is shown as the time required to cross 1km (1). The shapefile from the Centre National de l'Information Géo-Spatiale (CNIGS) was used (5) (available at https://data.humdata.org/dataset/hti-polbndl-adm1-cnigs-zip).

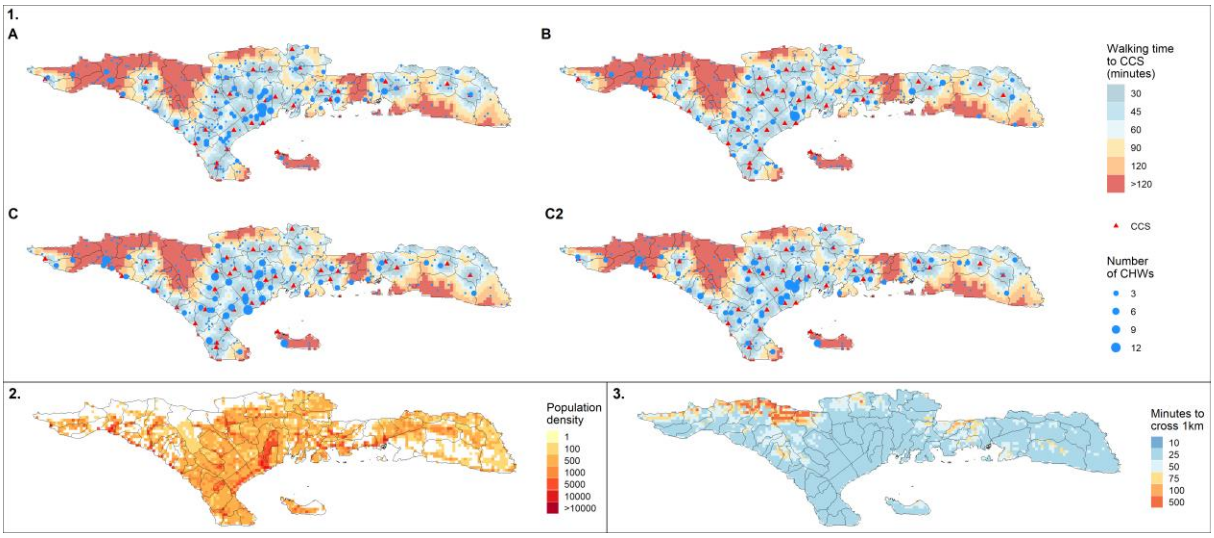

S1 Text Fig 8. Comparison of the placement scenarios in the Sud department. The four CHW placement scenarios (A, B, C and C2). CHW positions are indicated with blue dots, community health centres (CCS) are indicated with red triangles. The colored surface indicates the predicted walking time to the closest CCS using the methodology by Weiss and colleagues (1,2): difficult-to-reach areas, located more than 60 minutes walk from the nearest CCS, are shown in orange/red and areas with easier access (less than 60 minutes walk) are shown in blue. 2. For interpretability: prediction of population density in 2020 per square kilometre (3,4). 3. For interpretability: walking time friction surface by Weiss et al. is shown as the time required to cross 1km (1). The shapefile from the Centre National de l'Information Géo-Spatiale (CNIGS) was used (5) (available at https://data.humdata.org/dataset/hti-polbndl-adm1-cnigs-zip).

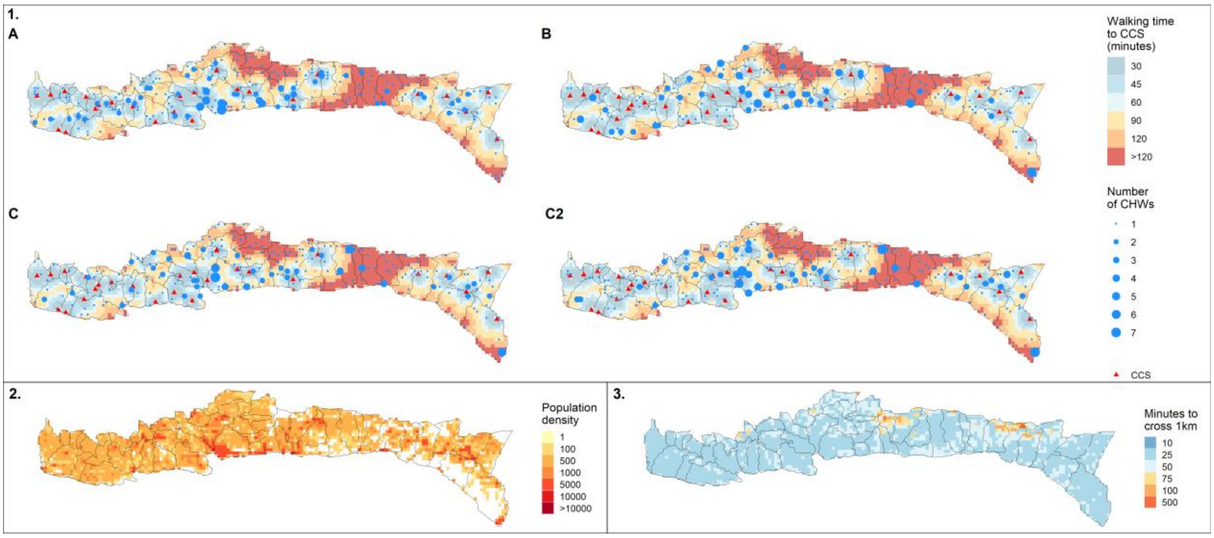

S1 Text Fig 9. Comparison of the placement scenarios in the Sud-Est department. The four CHW placement scenarios (A, B, C and C2). CHW positions are indicated with blue dots, community health centres (CCS) are indicated with red triangles. The colored surface indicates the predicted walking time to the closest CCS using the methodology by Weiss and colleagues (1,2): difficult-to-reach areas, located more than 60 minutes walk from the nearest CCS, are shown in orange/red and areas with easier access (less than 60 minutes walk) are shown in blue. 2. For interpretability: prediction of population density in 2020 per square kilometre (3,4). 3. For interpretability: walking time friction surface by Weiss et al. is shown as the time required to cross 1km (1). The shapefile from the Centre National de l'Information Géo-Spatiale (CNIGS) was used (5) (available at https://data.humdata.org/dataset/hti-polbndl-adm1-cnigs-zip).

References

1. Weiss DJ, Nelson A, Gibson HS, Temperley W, Peedell S, Lieber A, et al. A global map of travel time to cities to assess inequalities in accessibility in 2015. Nature [Internet]. 2018 Jan 18;553(7688):333–6. Available from: doi.org/10.1038/nature25181

2. Weiss DJ, Nelson A, Vargas-Ruiz CA, Gligorić K, Bavadekar S, Gabrilovich E, et al. Global maps of travel time to healthcare facilities. Nature Medicine [Internet]. 2020 Sep 28 [cited 2020 Nov 3];1–4. Available from: https://www.nature.com/articles/s41591-020-1059-1

3. Facebook, CIESIN. Facebook Connectivity Lab and Center for International Earth Science Information Network - CIESIN - Columbia University. 2016. High Resolution Settlement Layer (HRSL). Source imagery for HRSL © 2016 DigitalGlobe. Accessed 20.06.2019. 2016.

4. IHSI. Population totale, de 18 ans et plus. Ménages et densités estimés en 2015. [Internet]. Institut Haïtien de Statistique et d'Informatique - IHSI/Haiti; 2015 [cited 2019 Aug 12]. Available from: http://www.ihsi.ht/pdf/projection/Estimat_PopTotal_18ans_Menag2015.pdf

5. Centre National de l'Information Géo-Spatiale (CNIGS). Haiti - Subnational Administrative Boundaries [Internet]. 2013 [cited 2017 Oct 1]. Available from: https://data.humdata.org/dataset/hti-polbndl-adm1-cnigs-zipdataset/hti-polbndl-adm1-cnigs-zip
