# Supplementary material for: Improving access to care and community health in Haiti with optimized community health worker placement
Source: PLOS Glob Public Health. 2022 May 10;2(5):e0000167. doi: 10.1371/journal.pgph.0000167 (PMC10022239; doi:10.1371/journal.pgph.0000167)
Supplement: S2 Text — (PDF) [file pgph.0000167.s010.pdf]

S2 Text. Hypothetical total CHW numbers if travel time and population distribution were not considered

For comparison purposes, we also calculated the total numbers of CHWs required if only the population thresholds were accounted for, i.e. without considering travel time and population geographical distribution.

SCENARIO A	Urban	Rural	Metropolitan	Total
**Total population**	6710333	2651840	3086752	12448925
**Max. population per CHW (threshold)**	2500	1000	4000	
**Total number of CHWs on average (population/threshold)**	2684	2652	772	6108
**SCENARIO B**	**≤ 30 min from CCS**	**Urban**	**Rural**	**Total**
**Population**	4717476	5500715	2230733	12448924
**Max. population per CHW (threshold)**	0	2500	1000	
**Total number of CHWs on average (population/threshold)**	0	2200	2231	4431
**SCENARIO C**	**≤ 60 min from CCS**	**Urban**	**Rural**	**Total**
**Population**	8411101	2555038	1482785	12448924
**Max. population per CHW (threshold)**	4000	2500	1000	
**Total number of CHWs on average (population/threshold)**	2103	1022	1483	4608
**SCENARIO C2**	**Rural**	**Urban ≤ 60 from CCS**	**Urban > 60 min from CCS**	**Total**
**Population**	2671615	7222271	2555038	12448924
**Max. population per CHW (threshold)**	1000	4000	2500	
**Total number of CHWs on average (population/threshold)**	2672	1806	1022	5499

S2 Text. Table 1. Descriptive statistics on the population in urban, rural and difficult-to-reach areas. The average CHWs corresponds to the number of CHWs required if thresholds from Fig1 are applied on average population totals (i.e. without accounting for geographical population distribution and walking time). These totals are only provided for comparison purposes and were not used in the simulated scenarios.

Interestingly, if the same thresholds had been implemented on the average population totals without accounting for geographical population distribution and travel times (cf. S1 Text. Table 1), the required total numbers of CHWs would have been similar to or higher than four optimal scenarios retained (cf. Fig3, in the main text). This can be explained by two effects acting in opposite directions. On the one hand, accounting for the 60-minute travel time constraint increases the number of CHW required for the same number of individuals, because the population is not distributed evenly on the territory. On the other hand, to optimise the total number of CHWs, the algorithm sometimes assigns inhabitants from so-called rural areas to CHWs positioned in neighbouring urban areas, thus decreasing the total CHW numbers in rural areas. This effect is to be put in perspective, given that the algorithm always guarantees that the travel time for a CHW remains below 60 min and that rural and urban areas are defined only theoretically by population density.
