# Supplementary material for: Improving access to care and community health in Haiti with optimized community health worker placement
Source: PLOS Glob Public Health. 2022 May 10;2(5):e0000167. doi: 10.1371/journal.pgph.0000167 (PMC10022239; doi:10.1371/journal.pgph.0000167)
Supplement: S1 File — (HTML) [file pgph.0000167.s011.html]

Improving access to care and community health in Haiti with optimized community health worker placement


# Improving access to care and community health in Haiti with optimized community health worker placement

### Comparison of the four CHW placement scenarios at the national level

> Authors: Clara Champagne, Andrew Sunil Rajkumar, Paul Auxila, Giulia Perrone, Marvin Plötz, Alyssa Young, Samuel Bazaz Jazayeri, Harriet Napier, Arnaud Le Menach, Katherine Battle, Punam Amratia, Ewan Cameron, Jean-Patrick Alfred, Yves-Gaston Deslouches, Emilie Pothin

# CHW placement scenarios


Modelled CHW positions for the four CHW placement scenarios (A, B, C and C2) are indicated with colored circles. The positioning of community health centres (CCS) are indicated with blue crosses. The colored surface indicates the prediction of population density in 2020 per square kilometre (Facebook and CIESIN (2016)), the friction surface by Weiss et al. (2018), the predicted walking time to the closest CCS using the methodology by Weiss et al. (2020), and the urban and rural areas as predicted using the method from The World Bank (2017). The shapefile from the Centre National de l’Information Géo-Spatiale (CNIGS) (2013) was used (https://data.humdata.org/dataset/hti-polbndl-adm1-cnigs-zip).

The maximum walking time is fixed to 60 minutes in all scenarios. The maximum number of inhabitants per CHW is varied across the four scenarios.  
In scenario A, the entire territory is covered by CHWs, with a maximum population of 1000 per CHW in rural areas, 2500 in urban areas and 4000 in the metropolitan area.  
In Scenario B, only areas situated at more than a 30 minutes’ walk from a community health centre (CCS) are covered by CHWs, with a maximum population of 2500 per CHW in urban and metropolitan areas and 1000 in rural areas. In scenarios C and C2, the entire territory is covered by CHWs but the maximum population thresholds depend on the distance to the nearest CCS.  
In scenario C, less than a 60 minutes’ walk from a CCS, 4000 people are assigned to each CHW and more than a 60 minutes’ walk from a CCS, the maximum populations is 2500 per CHW in urban and metropolitan areas and 1000 in rural areas.  
Scenario C2 is similar to scenario C, except that the maximum population is 1000 in rural areas, whatever the distance to the closest CCS, and the 4000 threshold within a 60 minutes’ walk from a CCS is applied only for urban areas.

### Influence of parameter changes on scenario C

| Names | Meaning |
| --- | --- |
| Capacity -10% | maximum population per CHW decreased by 10% |
| Capacity +10% | maximum population per CHW increased by 10% |
| Time CHW-hh -10% | maximum walking time between CHW and households = 54 min |
| Time CHW-hh +10% | maximum walking time between CHW and households = 66 min |
| Time to CCS -10% | walking time to CCS to be considered as difficult-to-reach = 54 min |
| Time to CCS +10% | walking time to CCS to be considered as difficult-to-reach = 66 min |
| Urban min density -10% | minimum population density used to be define urban areas = 270 habitants / km^2 |
| Urban min density +10% | minimum population density used to be define urban areas = 330 habitants / km^2 |
| Urban min pop. -10% | minimum population size used to be define urban areas = 1800 habitants |
| Urban min pop. +10% | minimum population size used to be define urban areas = 2200 habitants |

## References

Centre National de l’Information Géo-Spatiale (CNIGS). 2013. “Haiti - Subnational Administrative Boundaries.” https://data.humdata.org/dataset/hti-polbndl-adm1-cnigs-zip.

Facebook, and CIESIN. 2016. “Facebook Connectivity Lab and Center for International Earth Science Information Network - CIESIN - Columbia University. 2016. High Resolution Settlement Layer (HRSL). Source Imagery for HRSL © 2016 DigitalGlobe. Accessed 20.06.2019.”

The World Bank. 2017. “Haitian Cities: Actions for Today with an Eye on Tomorrow.” http://documents.worldbank.org/curated/en/709121516634280180/pdf/122880-V1-WP-P156561-OUO-9-FINAL-ENGLISH.pdf.

Weiss, D. J., A. Nelson, H. S. Gibson, W. Temperley, S. Peedell, A. Lieber, M. Hancher, et al. 2018. “A Global Map of Travel Time to Cities to Assess Inequalities in Accessibility in 2015.” *Nature* 553 (7688): 333–36. https://doi.org/10.1038/nature25181.

Weiss, D. J., A. Nelson, C. A. Vargas-Ruiz, K. Gligorić, S. Bavadekar, E. Gabrilovich, A. Bertozzi-Villa, et al. 2020. “Global Maps of Travel Time to Healthcare Facilities.” *Nature Medicine*, September, 1–4. https://doi.org/10.1038/s41591-020-1059-1.
