# Supplementary material for: Improving access to care and community health in Haiti with optimized community health worker placement
Source: PLOS Glob Public Health. 2022 May 10;2(5):e0000167. doi: 10.1371/journal.pgph.0000167 (PMC10022239; doi:10.1371/journal.pgph.0000167)
Supplement: Attachment — Submitted filename: Response to reviewers_EN.docx [file pgph.0000167.s012.docx]

Dear Editor,

We thank the reviewers for their assessment on our manuscript. We have revised the manuscript to address the comments made and provided a detailed response to the review report below.

Sincerely,

Clara Champagne, on behalf of the authors

***Review Comments to the Author***

***Reviewer #1: This is an excellent piece of modelling based work, executed nicely. Work itself merits publication. However, I am afraid, in actual practice, this is never going to be useful in program setting, due to reasons know to every public health professional. I congratulate the team for coming up with such modelling work. At least it may inspire others to apply in some other settings which may be of use.***

We thank the reviewer for this positive feedback on our work
While this work is indeed focusing on the use of a model, it had programmatic value and can help program make data oriented decision. For example the model has been used during the elaboration of the national strategic plan on community health in 2020.

Making such modeling work more accessible can also help increase the use of data-informed evidence to support program decision making. To support this, we have added some interactive visualizations of the results (available in S1 File) and our computer code has been updated to enable the user to work with the open-source solver SCIP (and not only with the commercial solver Gurobi).

***Reviewer #2: Ensure the citations of authors is accurate; the authorship of a local Haitian author was unclear and the absence of this contributing author would seriously impinge on the credibility of the article, not to mention the ethics of global health research done to, and not with, local stakeholder engagement and ownership.***

We thank the reviewer for raising this important point. There was a mistake in the numbering of the affiliation indices of our colleagues from the Haitian Ministry of Health which has now been corrected.
Throughout this work there was ongoing communication and iteration between the modellers, the Ministry of Health (Ministère de la Santé et de la Population), the World Bank and the Global Fund. The collaboration with the national program helped ensure the correct assumptions were being used in the model, the placement scenarios were appropriate and more generally that to the specific needs of the Haitian community health programme were addressed. The results presented in the current manuscript are the outcome of this highly collaborative work. To better reflect the specific collaborative activities, we edited the following sentences (elements in italics are now added):

- in the results section:
  “Four scenarios of CHWs spatial optimization accounting for population distribution and travel time were selected *within an iterative process of interactions between modellers and decision makers to correspond as much as possible to the specific needs of the Haitian community health programme*”
- In the discussion section:
  “In this work, four scenarios for CHW geographical placement in Haiti were designed, accounting for population density, topography and the location of existing health facilities, *in concertation with the Ministry of Health*.”
